# Development of tCAP(N3): Affinity Peptide‐Aided, pH‐Triggered Strategy for Site‐Specific Native IgG Modification

**DOI:** 10.1002/chem.202503144

**Published:** 2025-12-24

**Authors:** Hiroko Kawakami, Abdur Rafique, Shugo Tsuda, Asaki Nagashima, Yukie Nohara, Naoki Dozono, Ken Sakamoto, Shun Masuda, Masato Kiyoshi, Hiroko Shibata, Akiko Ishii‐Watabe, Taku Yoshiya, Yuji Ito

**Affiliations:** ^1^ Graduate School of Science and Engineering Kagoshima University Kagoshima Japan; ^2^ Peptide Institute, Inc. Osaka Japan; ^3^ Division of Biological Chemistry and Biologicals National Institute of Health Sciences Kanagawa Japan; ^4^ Institute for Protein Research Osaka University Osaka Japan

**Keywords:** antibody, antibody drug ratio, cancer, payloads, toxic effects

## Abstract

Direct modification of native antibodies with affinity peptides remains a significant challenge, primarily because the affinity peptide often stays bound to the antibody and blocks its interaction with key receptors. In this study, we developed tCAP(N3), a novel traceless chemical conjugation method that employs an affinity peptide. tCAP(N3) can be stored for over a year at refrigerated temperature, as it contains an active ester precursor that can be spontaneously activated under neutral conditions. When tCAP(N3) was mixed with a target native IgG, Ac‐Lys(N_3_)‐Gly‐Gly was site‐selectively transferred onto Lys248, yielding divalently azidated IgG that can be further modified with DBCO compounds. The resulting antibody conjugates retained Fc receptor binding and antigen recognition comparable to the parent IgG.

## Graphical abstract

The tCAP(N_3_) reagent, featuring a stable and self‐activating precursor, enables site‐specific conjugation of native IgGs. This Fc‐affinity peptide approach selectively transfers an Ac‐Lys(N_3_)‐Gly‐Gly payload to Lys248, producing divalently azidated antibody while preserving its biological function. The incorporated azide groups serve as handles for efficient click chemistry with DBCO‐functionalized payloads, yielding well‐defined ADCs.

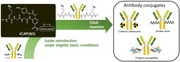

## 1 Introduction

Recently, chemical modification of antibodies has attracted considerable attention, as antibody‐based therapeutics have revolutionized the treatment of diseases such as cancer and autoimmune disorders. First‐generation antibody‐drug conjugates (ADCs) represented a breakthrough in cancer; however, significant complications emerged during clinical use [1]. Conventional methods for generating antibody conjugates typically involve random coupling strategies: either active esters reacting nonselectively with amino groups, or maleimide compounds reacting with thiol groups generated by partial antibody reduction. Recent antibody engineering techniques permit the insertion of additional residues—such as cysteine, unnatural amino acids, or tag peptides—that serve as anchoring points for site‐selective modification. However, producing such engineered antibodies remains labor‐intensive and costly [2]. Consequently, there has been growing interest in developing site‐specific modification methods that directly target native antibodies [3].

**FIGURE 1:**
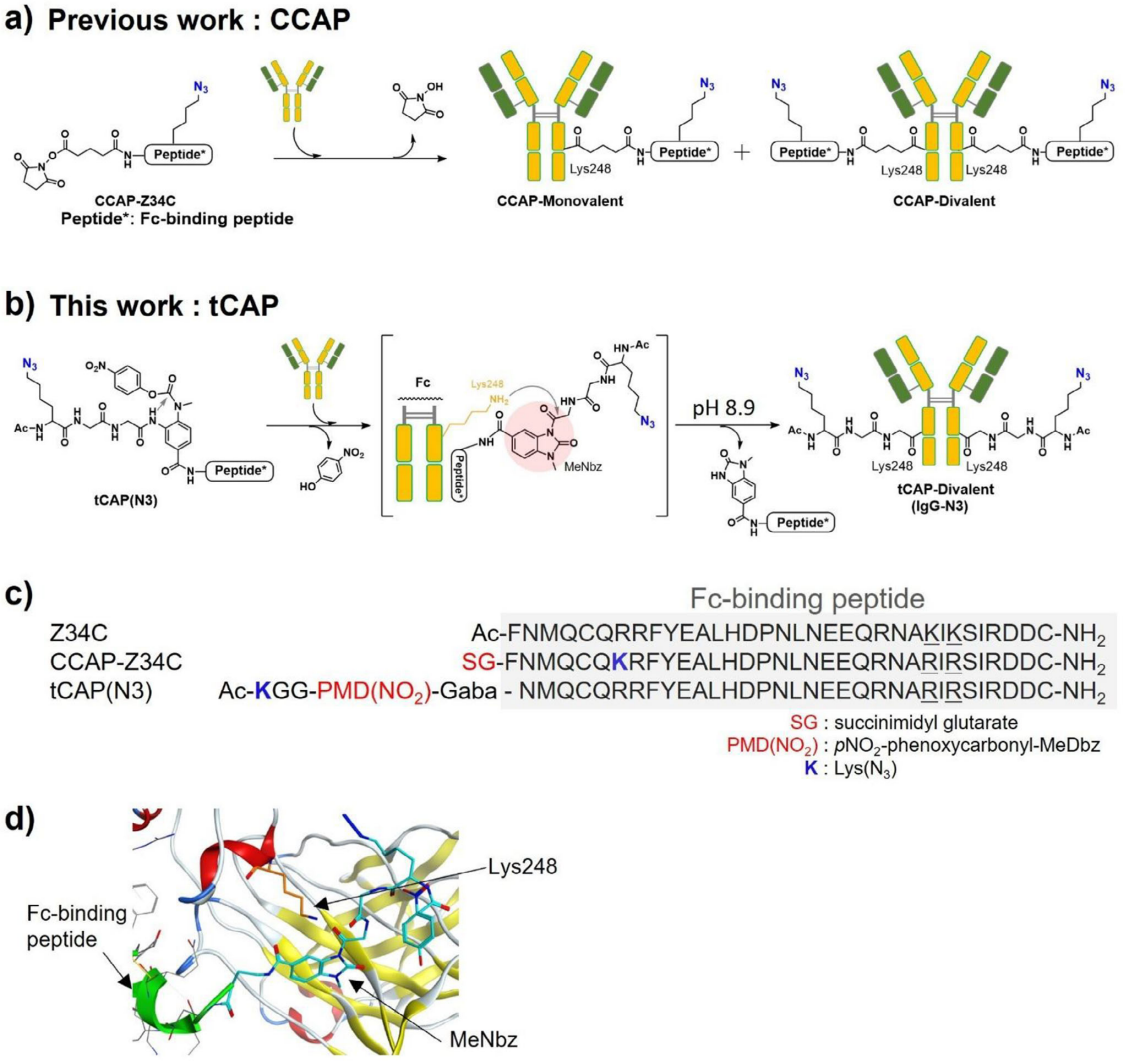
(a, b) Mechanism of antibody modification with CCAP‐Z34C and tCAP(N3). (c) Sequences of the Fc affinity peptide Z34C and its derivatives, CCAP‐Z34C and tCAP(N3). Blue K represents Lys(N_3_), and red SG indicates the succinimidyl glutarate (SG) moiety. (d) Model of human IgG_1_ complexed with the tCAP intermediate, highlighting the ε‐amino group of Lys 248 in the Fc region.

In 2019, our group developed a chemical conjugation by affinity peptide (CCAP) approach, which employs affinity peptides against human and rodent IgG [4, 5, 6, 7, 8, 9]. This approach enables site‐specific modification at defined Lys residues within the Fc region of the human IgG (Figure 1a). This methodology employs specially designed “CCAP reagents,” composed of three components: a payload, an affinity peptide, and an active ester. When a CCAP reagent is mixed with a native antibody, the affinity peptide bearing the payload becomes covalently attached at Lys248 through a reaction between the active ester and that specific Lys residue. Recently, reagents similar to CCAP—using Fc affinity peptides—have been reported for site‐specific IgG modification, generating antibody conjugates that retain a covalently linked affinity peptide [10, 11, 12, 13, 14, 15, 16]. However, such modifications have been shown to potentially inhibit the binding of IgG to FcRn and alter the physicochemical properties of the antibody's Fc region.

Building on this research, we envisioned a new type of antibody‐decorating reagents, arranged in the order “payload‐active ester‐affinity peptide,” which could transfer the payload onto the target antibody without leaving the peptide behind. This study reports a novel reagent, termed “tCAP,” consisting of a payload, active acyl donor, and affinity peptide in this sequence (Figure 1b). Unlike CCAP reagents, the tCAP system transfers the payload to the target antibody without leaving the affinity peptide bound to the Fc region, thereby producing a simpler antibody conjugate. Indeed, only a limited number of studies have recently reported similar reagents [17, 18, 19]. However, these approaches often require fine adjustment of stability and reactivity, likely because the active ester is positioned internally within the reagent, which hinders efficient payload transfer compared with traditional CCAP reagents that place the active ester at the terminus. To address this, we replaced the unstable active ester with its precursor, *p*NO_2_‐phenoxycarbonylated (Phoc)‐MeDbz [PMD(NO_2_)] [20, 21, 22, 23], which is stable under neutral pH yet can be spontaneously activated under mild alkaline conditions. This design extends shelf‐life and enhances the potential for broader applications. Additionally, this study describes the preparation of antibody conjugates with various payloads using the tCAP method and provides a biochemical and biological characterization of the resulting conjugates.

## 2 Results

### 2.1 Design of an Azide‐Transfer Reagent “tCAP(N3)”

For the affinity peptide of the tCAP molecule, we selected Z34C [24, 25], a broadly applicable affinity peptide derived from a fragment of protein A (Figure 1c). Efficient payload transfer requires precise spatial positioning of the PMD(NO_2_) unit. Therefore, we designed tCAP reagents using molecular simulations based on the structural data from the human IgG Fc‐Z34C co‐crystal complex (1oqo.pdb, Figure 1d). Based on these simulations, the N‐terminal Phe of Z34C was removed, and the optimal spacer length between the peptide and PMD(NO_2_) moiety was determined to be a C4 linker composed of γ‐aminobutyric acid (Gaba). Ultimately, we designed a candidate reagent, termed “tCAP(N3), ” containing Ac‐Lys(N_3_)‐Gly‐Gly as the payload to enable direct azidation of IgG.

### 2.2 In Situ Autoactivation of tCAP(N3) and Stability during Storage

tCAP(N3) was readily synthesized using standard Fmoc solid‐phase peptide synthesis (SPPS) (Scheme S1). Once synthesized, the autoactivation rate and storage stability of tCAP(N3) were first evaluated. As mentioned above, PMD(NO_2_) was adopted as a stable precursor of the active ester equivalent MeNbz. As shown in Figure S1, MeNbz‐containing active species were spontaneously generated under neutral conditions at room temperature. For example, MeNbz species were obtained within 1 h at pH 7 and within 20 min at pH 8. At pH 8.9, PMD(NO_2_) was rapidly converted to MeNbz, which was fully hydrolyzed within 1 h. These results confirmed the expected pH dependence of autoactivation. Furthermore, the stability of lyophilized tCAP(N3) was evaluated at −20 °C. As expected, tCAP(N3) could be stored for over one year without significant decomposition (Figure S2). By contrast, the reference compound CCAP‐Z34C, containing an active ester, was difficult to synthesize in high purity and exhibited significant degradation over time. These results demonstrate the superior stability of tCAP(N3) compared to CCAP reagents.

### 2.3 Preparation of the Azide‐Modified Trastuzumab (Tmab) by tCAP(N3)

**FIGURE 2:**
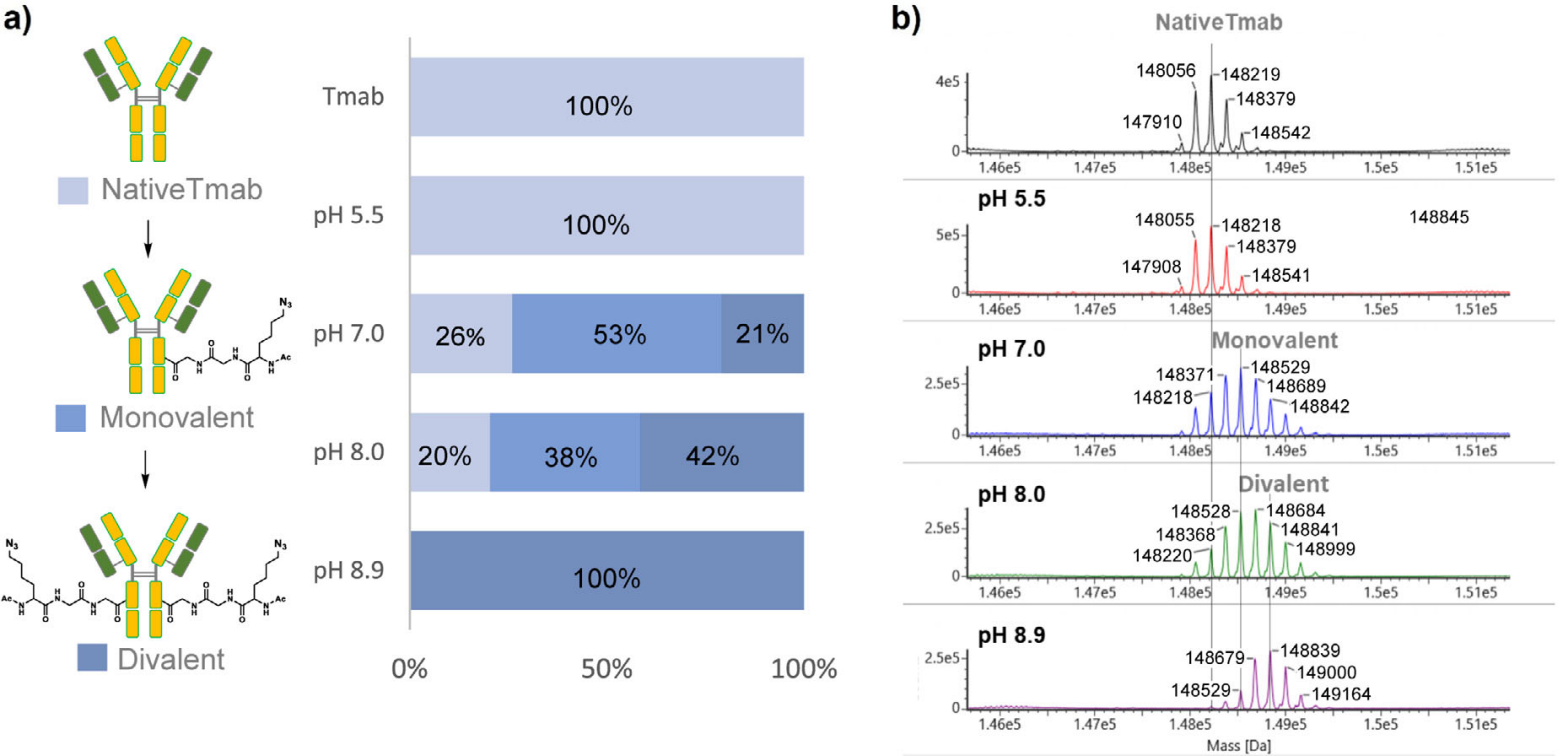
Effect of pH on Tmab modification by tCAP(N3). (a) Proportions of native, monovalently modified, and divalently modified antibodies at different pH values, determined by LC‐MS. (b) Representative LC‐MS chromatograms of Tmab after reaction with tCAP(N3) under different pH conditions.

**FIGURE 3:**
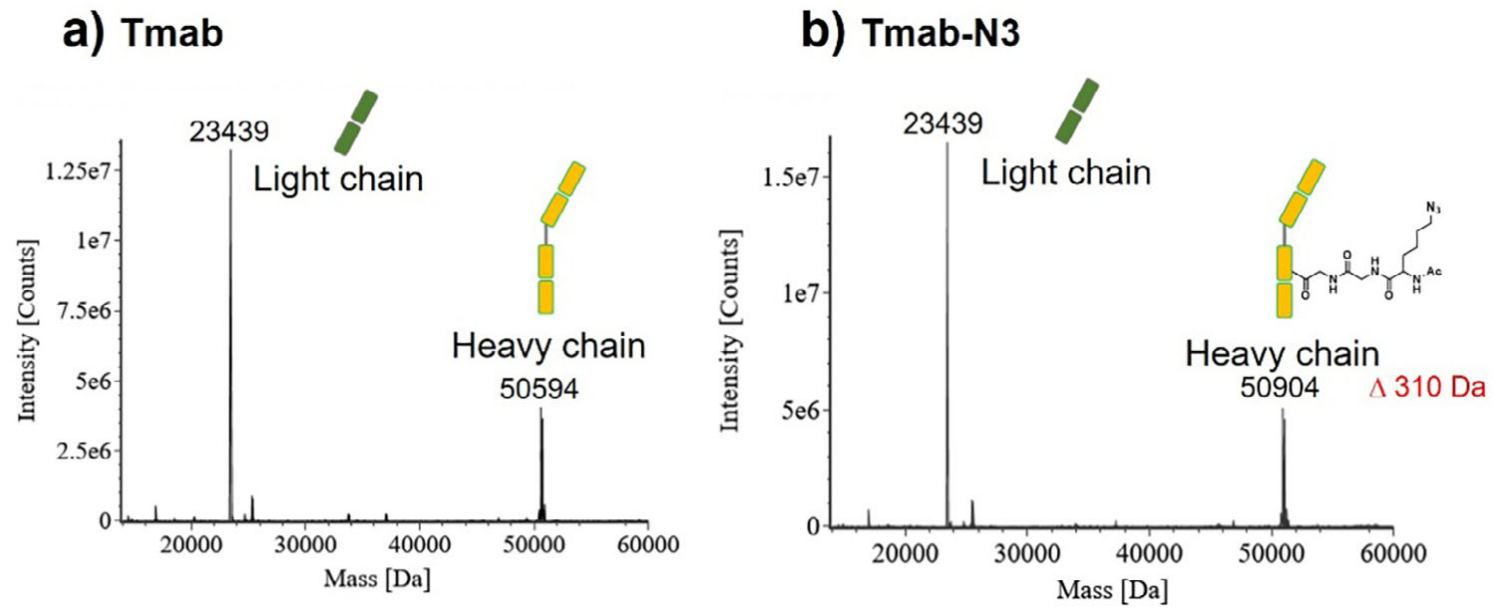
Mass spectra of Tmab under reducing conditions, before and after tCAP(N3) modification. (a) Native Tmab; (b) Azide‐modified Tmab (Tmab‐N3). A mass shift of approximately 310 Da appears only in the heavy chain, confirming site‐specific modification without affecting the light chain.

Antibody modification was performed by mixing Tmab with a fourfold molar excess of tCAP(N3) under various pH conditions (5.5–8.9) at room temperature. The reactions were evaluated by liquid chromatography‐mass spectrometry (LC‐MS), which revealed a mass increase of 310 Da per Ac‐Lys(N_3_)‐Gly‐Gly modification. As shown in Figure 2, the reaction was incomplete at pH 8.0 but proceeded to completion under more basic conditions (pH 8.9), yielding divalently azide‐modified Tmab (Tmab‐N3) with the appropriate mass increment of 620 Da (310×2). To verify that the tCAP(N3) specifically modified the Fc region of IgG, we analyzed native Tmab and Tmab‐N3 via mass spectrometry under reducing conditions. A mass increase of 310 Da was observed only in the heavy chain, whereas the light chain remained unchanged at 23, 439 Da, matching native Tmab (Figure 3). These results indicated that the modification was confined to the heavy chain. Additionally, site‐specific modification at Lys248 was confirmed by LC‐MS/MS analysis after enzymatic digestion (Figure S3). Moreover, affinity peptides were efficiently removed by acidic dialysis following the modification reaction, as described in the Supporting Information (Preparation of Azide‐KGG‐Modified Antibodies through tCAP Reaction). This procedure is effective because the affinity peptides lose their binding properties under acidic conditions.

### 2.4 Affinities of Tmab‐N3 Against Fc Receptors

Surface plasmon resonance (SPR) analysis was performed to compare the binding affinities of Tmab with Tmab‐N3 toward Fc receptors —FcγRI, FcγRIIa, FcγRIIb, FcγRIIIa, FcγRIIIb, and the neonatal Fc receptor (FcRn)— as summarized in **Table** **1** and Figure S4. Divalent modification at Lys248 with Ac‐Lys(N_3_)‐Gly‐Gly did not significantly alter the affinities for any of the Fc receptor proteins.

**TABLE 1:** Affinities of Tmab‐N3 for various Fc receptors, determined using SPR. The corresponding raw sensorgrams are shown in Figure S4.

Fc receptors	Tmab			Tmab‐N3		
	ka (1/Ms)	kd (1/s)	Kd (M)	ka (1/Ms)	kd (1/s)	Kd (M)
FcγRI	2.32 × 10^5^	1.24 × 10^−3^	5.34 × 10^−9^	2.11 × 10^5^	1.39 × 10^−3^	6.57 × 10^−9^
FcγRIIa	1.04 × 10^7^	1.06	1.01 × 10^−7^	6.11 × 10^6^	5.35 × 10^−1^	8.77 × 10^−8^
FcγRIIb	4.10 × 10^5^	1.10 × 10^−1^	2.68 × 10^−7^	4.32 × 10^5^	8.85 × 10^−1^	2.05 × 10^−6^
FcγRIIIa	3.89 × 10^5^	2.57 × 10^−2^	6.59 × 10^−8^	4.24 × 10^5^	2.77 × 10^−2^	6.53 × 10^−8^
FcγRIIIb	1.14 × 10^5^	4.66 × 10^−1^	4.08 × 10^−6^	9.50 × 10^4^	3.09 × 10^−1^	3.25 × 10^−6^
FcRn^*^	2.78 × 10^5^	1.58 × 10^−1^	5.68 × 10^−7^	6.14 × 10^5^	1.52 × 10^−1^	2.48 × 10^−7^

* Binding affinities to FcRn were analyzed using a biphasic fitting model; however, only the primary dissociation constant (*K*_D1_) values are reported.

### 2.5 Preparation of Antibody Conjugates From Tmab‐N3

**FIGURE 4:**
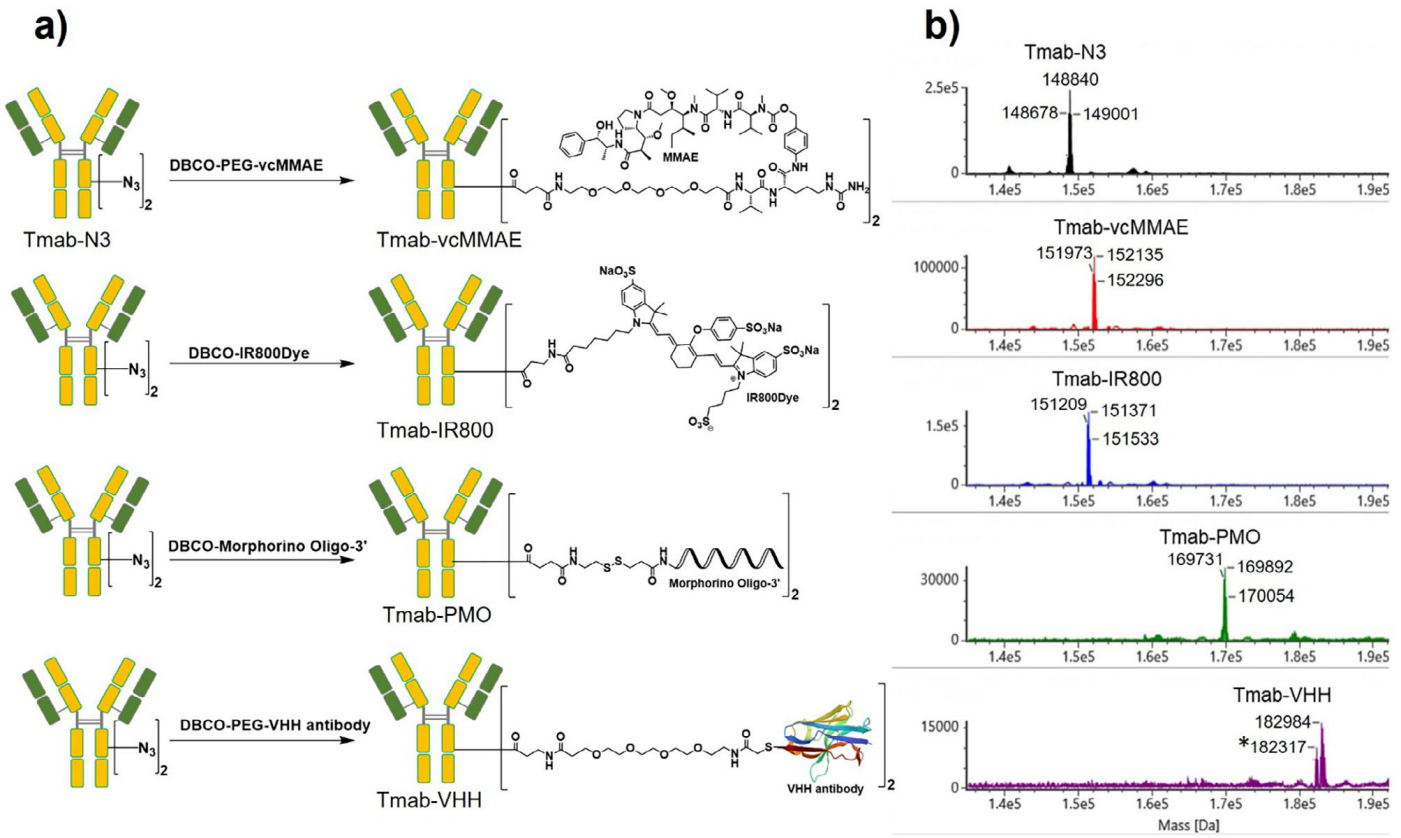
(a) Schematics of ADCs with different payloads generated via the tCAP reaction. (b) Mass spectra confirming successful conjugation of each ADC variant. *Presence of this smaller MS value is due to a glycoform variant of recombinant VHH protein.

Based on the positive outcomes above, as illustrated in Figure 4a, several antibody conjugates were prepared from Tmab‐N3 using strain‐promoted alkyne azide‐cycloaddition with a fivefold molar excess of DBCO‐functionalized payloads. We selected four heterogeneous payloads, chosen for their distinct properties and relevance to major biological applications: a monomethyl auristatin E derivative (vcMMAE) as an anticancer drug, IRDye‐800CW (IR800) as a relatively large fluorophore, hEx‐51 nucleic acid [26] as a phosphorodiamidate morpholino oligomer (PMO), and an FcαRI‐specific VHH antibody (VHH). The conjugation reaction was monitored by LC‐MS. Reactions with vcMMAE and IR800 reached completion within approximately 3 h, whereas those with PMO and VHH required up to 20 h. Despite these differences in kinetics, all conjugation reactions achieved high overall efficiency. Mass spectrometry analysis confirmed the formation of bivalently modified conjugates, with each displaying the expected mass increase corresponding to two payloads per Tmab‐N3 (Figure 4b). The purity of these antibody conjugates was further confirmed via size‐exclusion chromatography, with all products demonstrating purity levels greater than 95%. (Figure S5).

### 2.6 Determination of Half Maximal Inhibitory Concentration (IC_50_) Values of ADCs Using Competitive Enzyme‐Linked Immunosorbent Assay (ELISA) for Monitoring Antigen Binding

**FIGURE 5:**
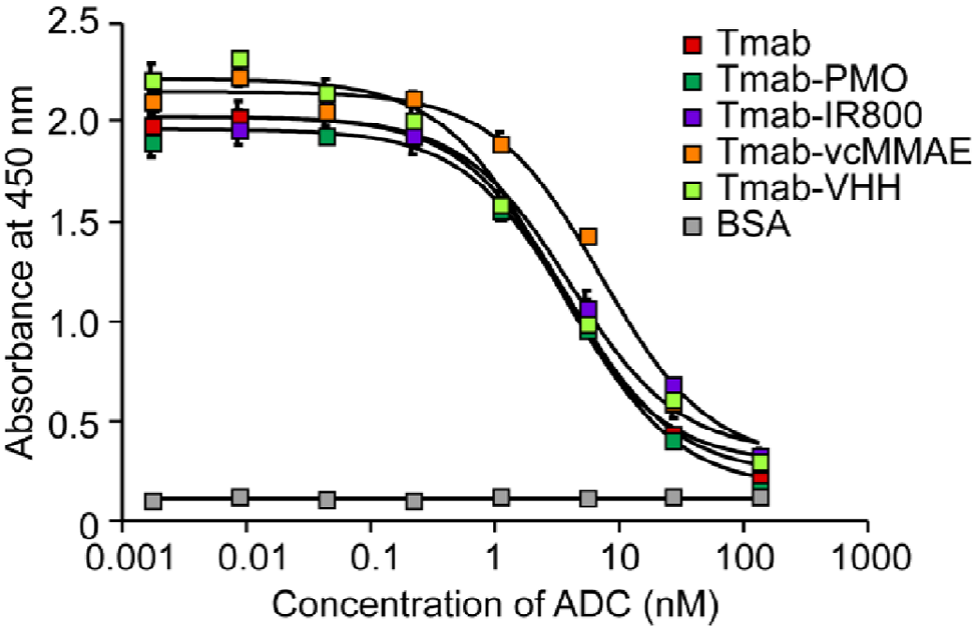
Competitive ELISA of Tmab‐based ADCs with different payloads. The inhibitory activity of each ADC was evaluated by measuring its ability to compete with biotinylated Tmab for binding to immobilized HER2 protein. Reduced signal intensity indicates effective competition with HER2 binding, demonstrating preserved antigen recognition after conjugation. The IC_50_ values, determined via competitive ELISA, were as follows: native Tmab (3.9 nM), Tmab‐IR800 (3.8 nM), Tmab‐PMO (4.2 nM), Tmab‐vcMMAE (6.6 nM), and Tmab‐VHH (2.5 nM). All experiments were performed with n = 3, and error bars represent the standard deviation from the mean.

The antigen‐binding IC_50_ values of each ADC were determined using a competitive ELISA. The assay employed a serial dilution of ADC solutions. Biotinylated Tmab (5 ng; 672 pM) was mixed with each diluted ADC solution and added to wells coated with immobilized antigen (Figure 5). Most ADCs exhibited IC_50_ values comparable to native Tmab, whereas Tmab‐MMAE showed approximately a twofold increase, indicating a modest reduction in binding affinity upon conjugation.

### 2.7 Binding Properties of Prepared ADCs Analyzed by Flow Cytometry

The antigen‐binding ability of the prepared ADCs was evaluated by flow cytometry using HER2‐positive SKBR3 and HER2‐negative C6 cells. All ADCs bound obviously to SKBR3 cells, whereas negligible binding was observed with C6 cells (Figure S6). These results confirmed that all ADCs retained strong and specific HER2 binding, demonstrating preservation of antigen recognition after conjugation.

### 2.8 Evaluation of FcRn Binding of ADCs

**FIGURE 6:**
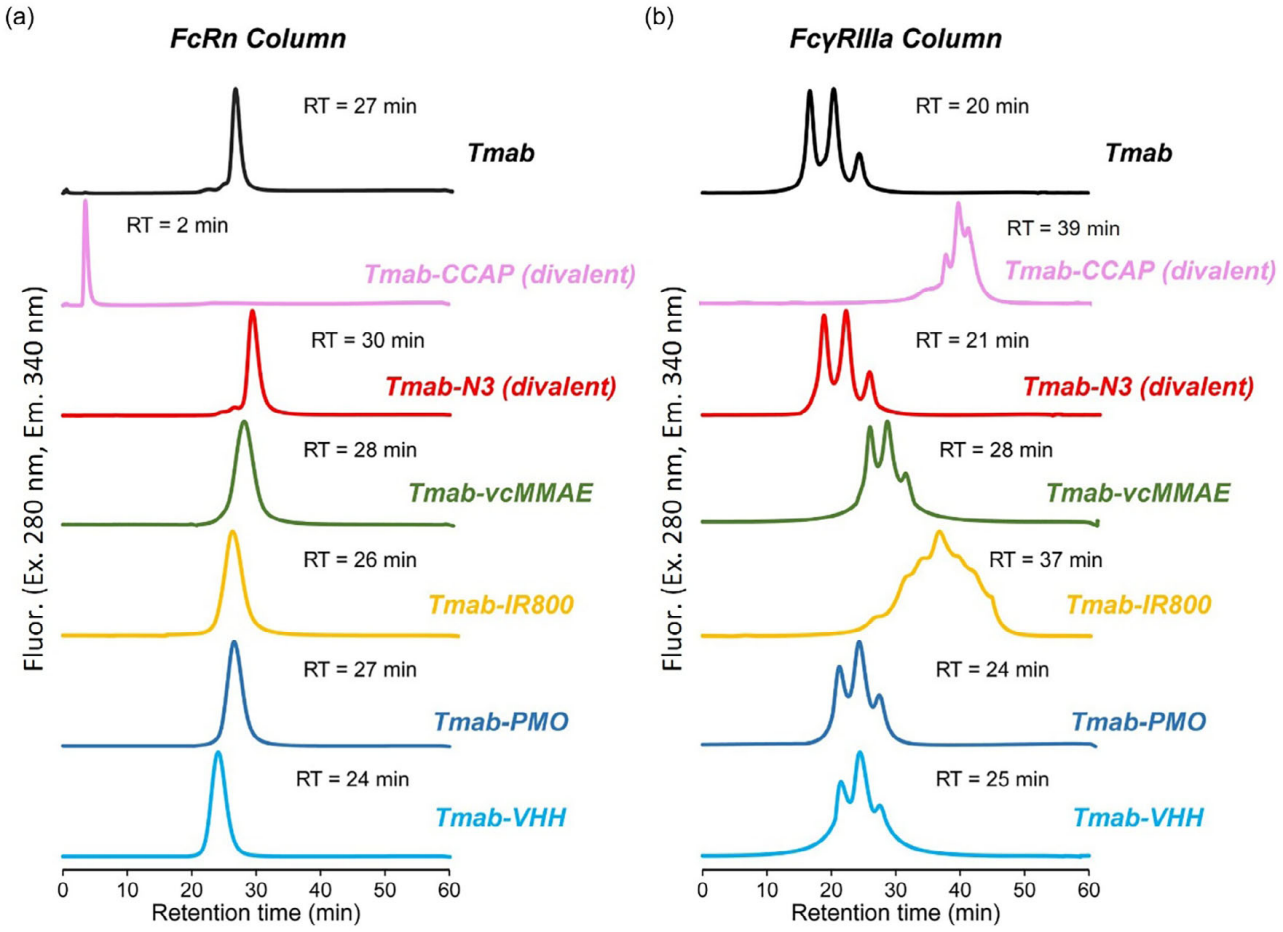
Binding profiles of ADCs with FcRn and FcγRIIIa. Prepared ADCs were injected into affinity columns immobilized with either (a) FcRn or (b) FcγRIIIa. Elution was performed using receptor‐specific pH gradients and monitored by fluorescence (excitation at 280 nm, emission at 340 nm). Elution profiles indicate the relative binding affinity of each ADC to its respective Fc receptor.

To satisfy the necessary requirements of a precise ADC, the FcRn binding capacity was evaluated using chromatographic techniques, as neonatal FcRn plays a key role in protecting IgG from catabolism within endosomes and extending its half‐life in serum [27]. Each ADC was applied to an affinity column conjugated with recombinant FcRn and eluted using pH 6.5–8.5 to assess its FcRn binding capacity (Figure 6a). Tmab was eluted at 27 min. With the exception of Tmab‐CCAP (divalent), previously reported to lack FcRn binding ability [4], all Tmab‐derived conjugates eluted with similar retention times of 24–30 min. These findings indicated that FcRn binding affinity was preserved in all ADCs. Therefore, these ADCs are expected to exhibit pharmacokinetic profiles similar to the parent IgG, owing to their retained FcRn‐mediated recycling.

### 2.9 Evaluation of FcγRIIIa Binding of ADCs

Additionally, the FcγRIIIa binding function was evaluated using a chromatographic technique described in a previous study [28]. This interaction is critical for mediating antibody‐dependent cellular cytotoxicity (ADCC) [28, 29]. As shown in Figure 6b, Tmab eluted as three distinct peaks, corresponding to Fc glycoforms with different affinities for FcγRIIIa. The main peak of Tmab eluted at 20 min, whereas Tmab‐N3 eluted at 21 min. Most conjugates, except for Tmab‐IR800, eluted more slowly (24‐–‐28 min). This result indicates that the conjugates exhibited slightly enhanced binding to FcγRIIIa than native Tmab. Notably, Tmab‐IR800 eluted considerably later (37 min), a retention time comparable to that of Tmab‐CCAP (divalent), suggesting an unusually strong interaction with FcγRIIIa. While the underlying mechanism for this observation remains unclear, nonspecific interactions with the column resin, possibly owing to alterations in the physicochemical properties of Tmab‐IR800, cannot be ruled out. Therefore, further investigation is required to clarify the potential impact of this finding on ADCC activity.

### 2.10 Cell Cytotoxicity Assay of ADCs

**FIGURE 7:**
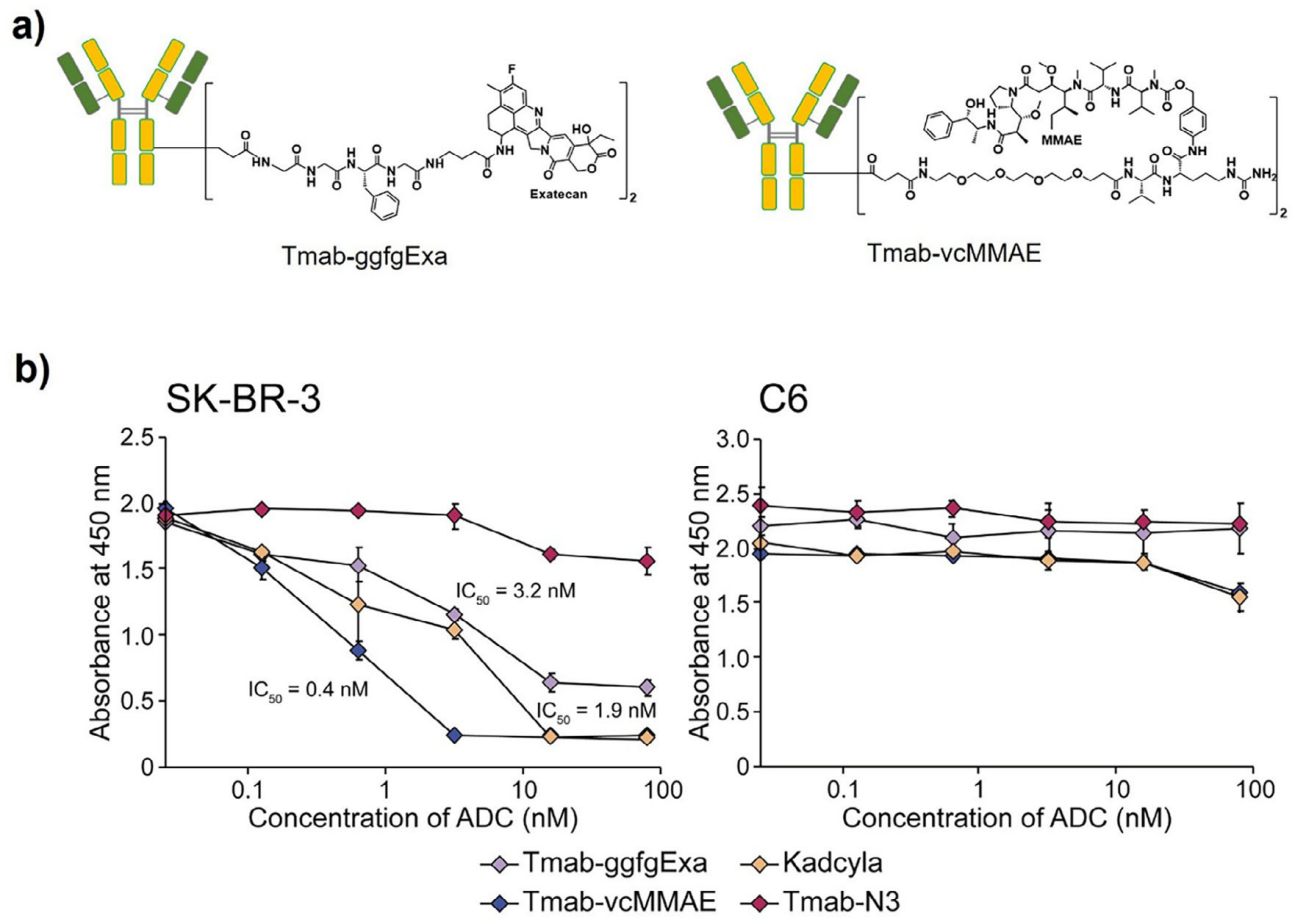
(a) Schematic of ADCs conjugated with exatecan and MMAE using the tCAP(N3) method. (b) Cytotoxic activities of the ADCs against HER2‐positive SKBR3 and HER2‐negative C6 cells. All experiments were performed with n = 3, and error bars represent the standard deviation from the mean.

The cytotoxic activity of ADCs prepared using the tCAP method (drug‐to‐antibody ratio (DAR) = 1.9) was evaluated using HER2‐positive SKBR3 cells (Figure 7). Besides Tmab‐vcMMAE, an exatecan‐containing ADC (Tmab‐ggfgExa) was synthesized and evaluated, reflecting recent trends in ADC development, as exatecan alone has relatively low cytotoxicity.[30, 31] Both ADCs exhibited potent cytotoxic effects, consistent with previous reports [32, 33], indicating that ADCs generated by the tCAP method effectively retain anticancer activity.

## 3 Discussion

In 2019, our group developed the CCAP method, which enables site‐specific chemical modification of native IgGs at Lys248 using Fc affinity peptides [4, 7, 8, 9]. However, because the affinity peptide remained covalently attached at this position, the resulting antibody conjugates lost their natural interaction with the FcRn [4]. As FcRn binding is essential for antibody recycling *in vivo*, antibodies bearing divalent affinity peptides on both Fc regions exhibited a reduced half‐life. To address this limitation, we occasionally isolated monovalent CCAP‐modified antibodies for *in vivo* studies [6]. However, separating monovalent antibody conjugates from mixtures of native and divalent forms was challenging. Similarly, in the AJICAP method developed by our group and Ajinomoto [5, 15], the affinity peptides are removed from the antibody through a secondary step, such as partial reduction after the initial conjugation. To simplify the production of FcRn‐compatible antibody conjugates, we envisioned a new class of reagents arranged in the order “payload‐active ester‐affinity peptide.” This design enables direct payload transfer onto the target antibody without leaving the affinity peptide covalently attached to its surface.

Fc affinity peptides used in such site‐specific IgG modification can be classified into two categories based on their origin [34]. The first consists of protein A‐derived fragments, such as the Z34C peptide (34 residues), which contains intramolecular disulfide bonds [24, 25]. The second includes human antibody‐specific peptides, such as FcIII and IgG‐BP, isolated from random peptide libraries [4, 35]. These peptides are generally shorter and contain a single disulfide bond. We previously confirmed that Lys248 of IgG can be selectively modified using both classes of affinity peptides [4, 7]. In this study, we adopted a Z34C derivative to ensure broad applicability.

**SCHEME 1:**
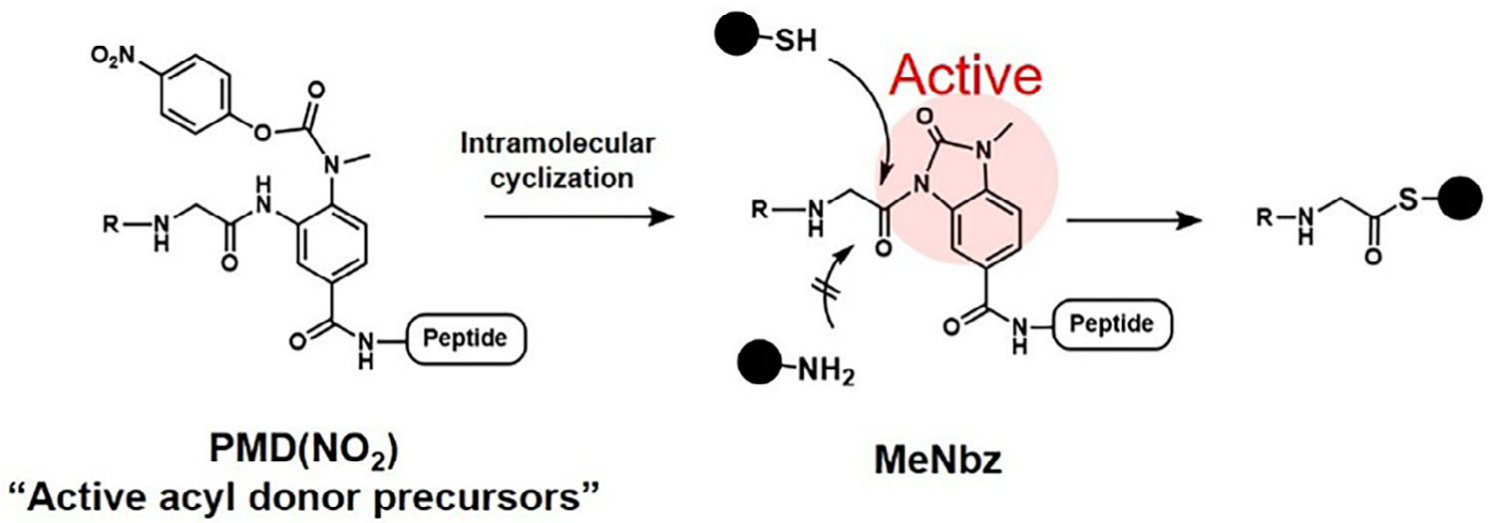
Autoactivation of PMD(NO_2_)‐containing peptides to MeNbz‐containing peptides, which readily react with strong nucleophilic thiols but not with weaker nucleophilic species such as amines.

Active esters, which are essential for IgG modification, often exhibit poor storage stability. To overcome this, we introduced a precursor structure for the acyl donor. Generally, the reactivity and stability of active esters are inversely related; highly reactive active esters tend to be unstable. Therefore, fine‐tuning both properties is essential when designing peptide‐based tools with an active ester [16, 17]; however, this optimization has practical limitations. To address this issue, we employed methyldiaminobenzoate (MeDbz) chemistry developed by Drs. Dawson and Blanco‐Canosa group [21]. MeDbz‐containing peptides can be synthesized using conventional Fmoc‐based SPPS and subsequently activated to yield MeNbz‐containing peptides, which function as active acyl donors. Additionally, N‐acylated MeDbz intermediates, such as PMD(NO_2_), can be isolated as active acyl donor precursors. Under neutral or mild alkaline pH conditions, these intermediates undergo intramolecular cyclization to spontaneously generate active acyl donor species in situ, a process known as pH‐triggered activation [22]. PMD(NO_2_) was first reported in N‐to‐C native chemical ligation (NCL) for protein synthesis. Thus, to develop stable and reactive antibody‐modifying reagents, we selected PMD(NO_2_) as a self‐activatable unit, as it provides both long‐term shelf stability and sufficient reactivity. While MeNbz‐containing peptides are typically used as acyl donors for nucleophilic thiol groups during NCL (Scheme 1) [36], we hypothesized that specific amino groups on native IgG, although weaker nucleophiles, could attack the MeNbz moiety through a proximity effect [37, 38] induced by the specific binding of the affinity peptide to the Fc region. We proposed that the relatively low electrophilicity of MeNbz underlies the observed site specificity. Notably, simple mixing of native Tmab with tCAP(N3) yielded divalently modified Tmab‐N3 within 30 min at pH 8.9, whereas the reaction did not proceed to completion at pH 8 or lower. As in situ autoactivation of PMD(NO_2_) occurs below pH 8 (Figure S1), the slower modification under these conditions was likely due to reduced nucleophilicity of the Lys248 side‐chain amino group, whose pKa is approximately 9.

Tmab‐N3 was further modified using standard DBCO‐mediated conjugation methods to generate four antibody conjugates: Tmab‐vcMMAE, Tmab‐IR800, Tmab‐PMO, and Tmab‐VHH. SPR analysis confirmed that the Fc receptor binding affinities of Tmab‐N3 were compared to native Tmab. Furthermore, FcRn affinity high‐performance liquid chromatography (HPLC) (Figure 6a) demonstrated that the antibody conjugates interacted with FcRn similarly to native Tmab, which plays a critical role in antibody recycling and half‐life maintenance in serum. These observations are consistent with the structural expectation that lies outside the FcRn‐Fc interaction interface (Figure S7). Conversely, FcγRIIIa affinity HPLC (Figure 6b) indicated that antibody modification altered FcγRIIIa interactions, as evidenced by prolonged column retention compared to the unmodified Tmab. As previously reported, CCAP modification of IgG stabilizes the Fc structure, reduces its molecular motion, and thereby enhances FcγRIIIa affinity [9]. Therefore, the delayed elution of Tmab‐IR800, which resembles that of divalent Tmab‐CCAP, may reflect a similar modification‐induced effect; however, a more detailed investigation is still required. Antigen recognition was validated by ELISA and cell‐based fluorescence‐activated cell sorting assays, both of which showed no significant impairment after divalent modification. These results suggest that tCAP(N3)‐derived antibody conjugates generally retain their functional properties in biological assays. Nevertheless, the biological activity of each conjugate should be evaluated individually. The cytotoxicity of several antibody conjugates was also experimentally confirmed. Further development of this approach may enable the design of new reagents targeting alternative modification sites [39, 40].

This study focused on Tmab modification because it represents a widely used human IgG_1_ antibody. As the affinity peptide in tCAP(N3) is derived from a protein A fragment and exhibits strong binding to multiple IgG subclasses, including human IgG_1_, IgG_2_, IgG_4_, mouse IgG_2a_, IgG_2b_, IgG_3_, and Rabbit IgG, these antibodies could be modified similarly. Ongoing work in our group is applying tCAP(N3) to several IgG antibodies, and the biological results will be reported in due course.

## 4 Conclusion

tCAP(N3) can be synthesized using standard Fmoc SPPS and stored under refrigerated conditions for over one year. Additionally, tCAP(N3) enables the Lys248‐selective incorporation of multiple payloads while largely preserving Fc functionality and antigen recognition. Therefore, this Fc‐directed strategy provides a precise and effective approach for IgG modification, offering controlled DAR while maintaining monoclonal antibody function. These characteristics are essential for the development of ideal ADCs. Overall, this strategy offers a versatile platform for advancing targeted antibody‐based therapeutics and diagnostics.

## Author Contributions

HK and AR designed experiments, analyzed data, and wrote the original manuscript. HK, ST, YN, KS, and SM synthesized peptides and evaluated chemical stability. AR, CU, AN, and ND performed antibody conjugation, binding assay, and cellular assay. MK, HS, and AI‐W designed, performed, and evaluated the SPR study. TY and YI designed the study, supervised the research, and edited the manuscript. All authors reviewed the manuscript and accepted the final version.

## Conflicts of Interest

Yuji Ito, Taku Yoshiya, and Shugo Tsuda have a patent PCT/JP2022/033713 pending to Kagoshima University and Peptide Institute, Inc. The other authors declare that they have no known competing financial interests or personal relationships that could have appeared to influence the work reported in this paper.

## Supporting information



Experimental details and supporting figures are provided in the Supporting Information with additional references [41].
**Supporting File**: chem70627‐sup‐0001‐SuppMat.docx.
