# Supplementary material for: Development of tCAP(N3): Affinity Peptide‐Aided, pH‐Triggered Strategy for Site‐Specific Native IgG Modification
Source: Chemistry. 2025 Dec 24;32(6):e03144. doi: 10.1002/chem.202503144 (PMC12887602; doi:10.1002/chem.202503144)
Supplement: Supplementary file 1 — Experimental details and supporting figures are provided in the Supporting Information with additional references [41]. Supporting File: chem70627‐sup‐0001‐SuppMat.docx. [file CHEM-32-e03144-s001.docx]

Supporting Information

Development of tCAP(N3): Affinity Peptide-Aided, pH-Triggered Strategy for Site-Specific Native IgG Modification

Hiroko Kawakami^[a, b]#^, Abdur Rafique^[a]#^, Shugo Tsuda^[b]^, Chikako Ueno^[a]^, Yukie Nohara^[b]^, Asaki Nagashima^[a]^, Ken Sakamoto^[b]^, Naoki Dozono^[a]^, Shun Masuda^[b]^, Masato Kiyoshi^[c]^, Hiroko Shibata^[c]^, Akiko Ishii-Watabe^[c]^, Taku Yoshiya^[b, d]*^ and Yuji Ito^[a]*^

[a] Graduate School of Science and Engineering, Kagoshima University, Kagoshima, Japan

[b] Peptide Institute, Inc., Osaka, Japan.

[c] Division of Biological Chemistry and Biologicals, National Institute of Health Sciences, Kanagawa, Japan

[d] Institute for Protein Research, Osaka University, Osaka, Japan

^#^These authors contributed equally to this work.

*Email: t.yoshiya@peptide.co.jp

*Email: yito@sci.kagoshima-u.ac.jp

**[Scheme and Figures]**


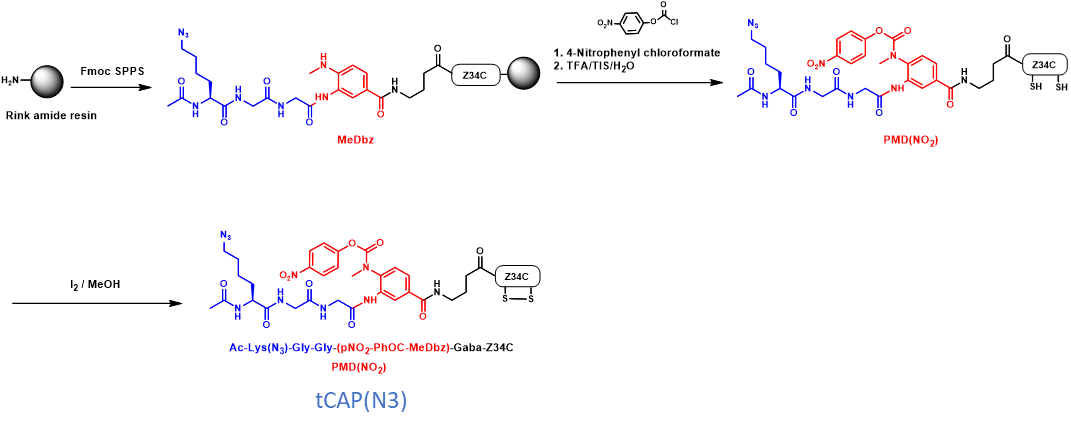


**Scheme S1.** Solid-phase peptide synthesis of tCAP(N3).


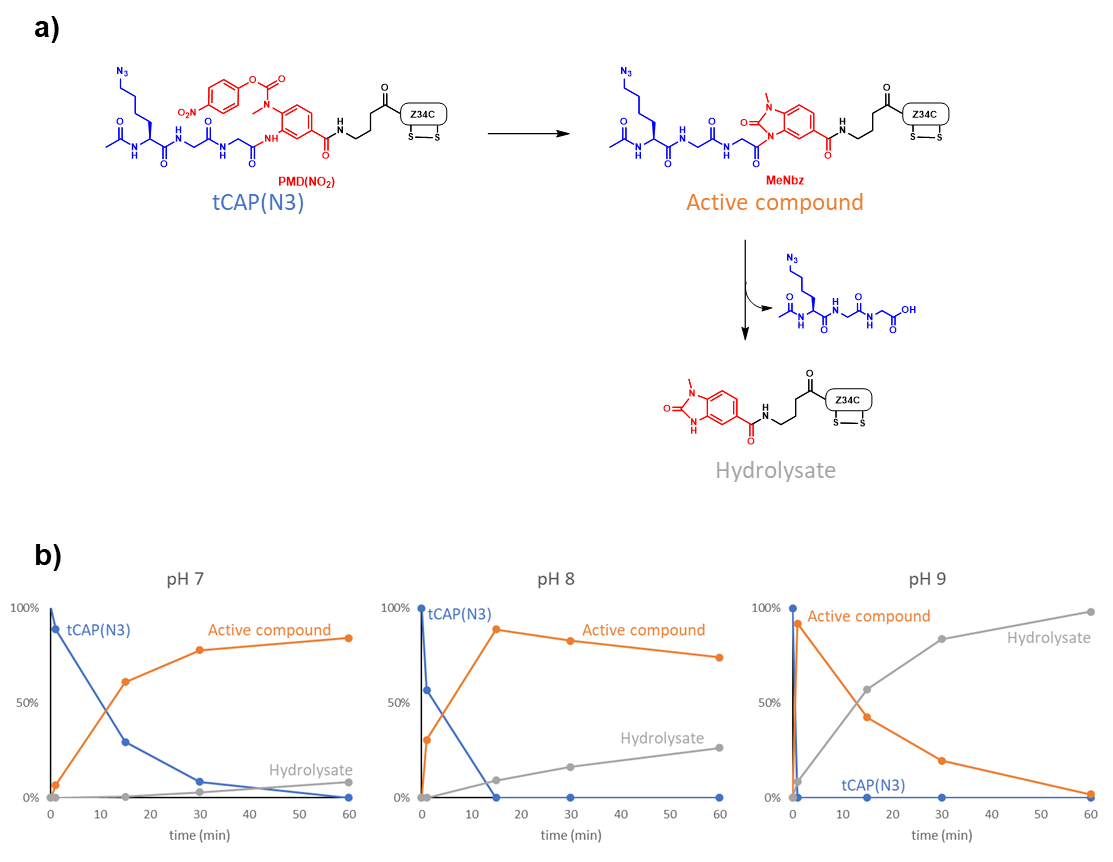


**Figure S1.** Autoactivation of tCAP(N3) under different pH conditions at room temperature.


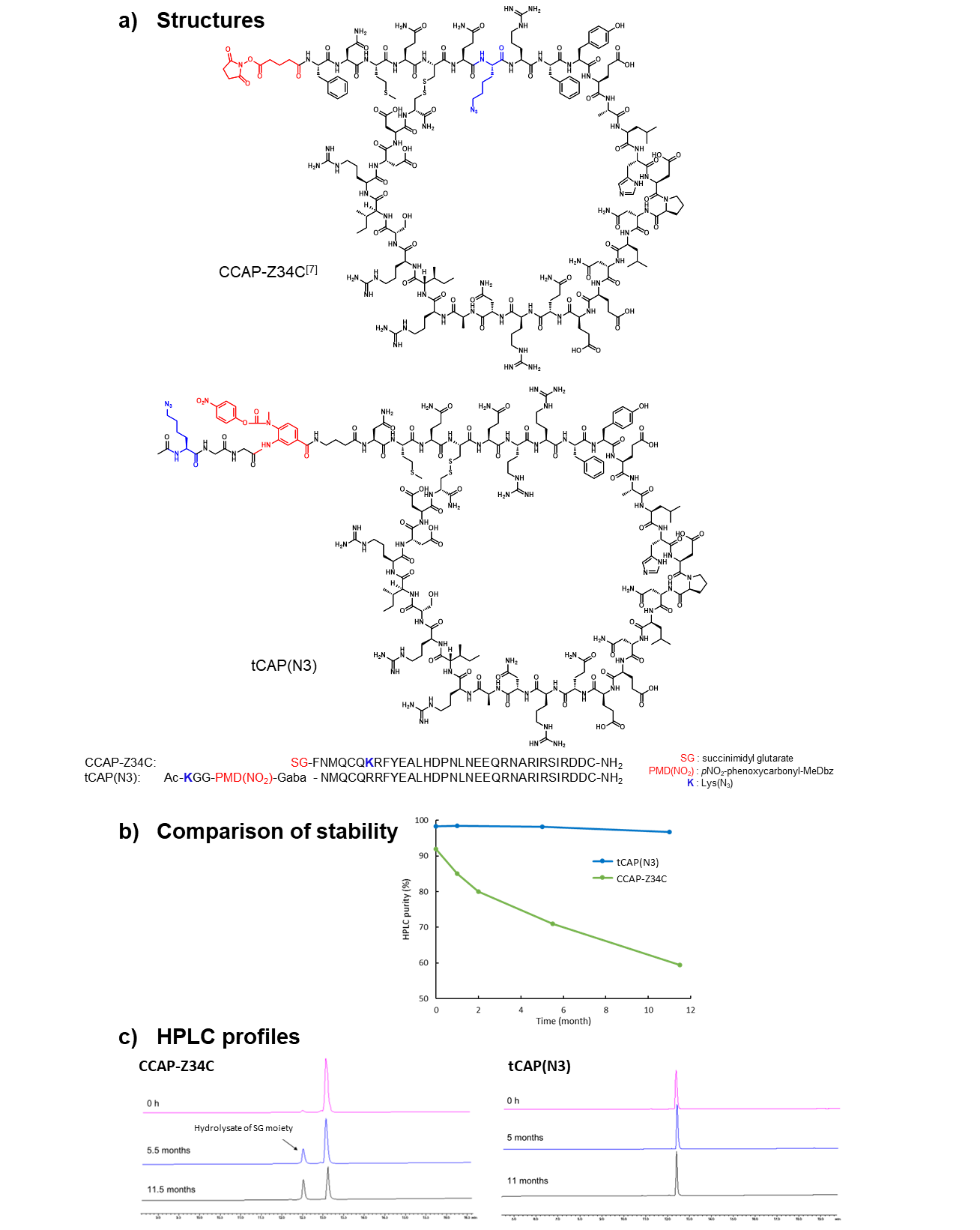


**Figure S2.** Structures of CCAP-Z34C and tCAP(N3) and their stability comparison: (a) structures of CCAP-Z34C and tCAP(N3), (b) time course of HPLC purity, and (c) raw HPLC traces. CCAP-Z34C was prepared according to Ref 7 in the manuscript.

**Figure S3.** LC-MS/MS analysis of Tmab-N3 after enzymatic digestion, indicating Lys248-selective modification. (A) Comparison of LC profile between Tmab-N3 (upper) and Tmab (lower). (B) MSMS results of T19-21 peak in Tmab-N3. (C) Sequencing results of T19-21 peak around Lys248.

**Figure S4.** Affinity measurements of Tmab–N3 toward FcRn and Fcγ receptors by SPR analysis.

**Figure S5.** Purity analysis of Tmab-based antibody–drug conjugates (ADCs) with heterogeneous payloads by SEC–HPLC**.** Size-exclusion chromatography was performed using an ENrich SEC 650 column (10 × 300 mm, Bio-Rad) equilibrated with 0.1 M Tris-HCl containing 0.15 M NaCl (pH 7.0). The chromatograms illustrate the separation profile of the conjugated species, with a predominant peak corresponding to the main conjugated product and minor peaks (<3%) representing dimers or potential by-products.

**Figure S6.** Antigen binding analysis of Tmab-based ADCs by flow cytometry. Flow cytometric analysis was performed using HER2-positive SKBR3 cells (upper panel) and HER2-negative C6 cells (lower panel).

**Figure S7.** Model structure of the Fc/FcRn complex modified at Lys248 with Ac–Lys(N₃)–Gly–Gly, constructed using the molecular modeling software MOE (Chemical Computing Group) based on the crystal structure of the Fc/FcRn complex (PDB ID: 4N0U). The modification was introduced at the ε-amino group of Lys248 in the Fc region.

**[Methods]**

**General Information on Chemical Synthesis**

All reagents and solvents were obtained from Peptide Institute, Inc. (Osaka, Japan), FUJIFILM Wako Pure Chemical Corporation (Osaka, Japan), Tokyo Chemical Industry Co., Ltd. (Tokyo, Japan), Nacalai Tesque, Inc. (Kyoto, Japan), Watanabe Chemical Industries, Ltd. (Hiroshima, Japan), and Merck KGaA (Darmstadt, Germany). Preparative HPLC was carried out on a Shimadzu liquid chromatograph (Model LC-8A, Kyoto, Japan), equipped with a YMC-Pack ODS-A (30 x 250 mm). The solvent system consisted of 0.1% TFA in H_2_O and 0.1% TFA in CH_3_CN, operated at a flow rate of 20 mL min^-1^ with detection at 220 nm. Analytical HPLC was performed on a Shimadzu liquid chromatograph (Model LC-10A, Kyoto, Japan) with a YMC-Pack ODS-A column (4.6 x 150 mm). The solvent system was the same (0.1% TFA in H_2_O and 0.1% TFA in CH_3_CN) and was run at a flow rate of 1 mL min^-1^ at 40 °C, with detection at 220 nm. Low-resolution mass spectrometry (MS) was performed using an Agilent InfinityLab LC/MSD detector coupled to an Agilent 1260 Infinity II HPLC system. For deconvolution, the observed masses (most abundant) were derived from the experimental m/z values for each protonation state of the target peptide.

Automated Fmoc SPPS was performed on a PurePep Chorus peptide synthesizer (Gyros Protein Technologies, Sweden). Peptide chain elongation employed the coupling protocol Fmoc-amino acid/DIC/OxymaPure. The following side-chain-protected amino acids were used: Arg(Pbf), Asn(Trt), Asp(OMpe), Asp(OtBu), Cys(Trt), Glu(O*t*Bu), Gln(Trt), His(Trt), Lys(Boc), Ser(*t*Bu), Thr(*t*Bu), Trp(Boc), and Tyr(*t*Bu). Fmoc-MeDbz was manually coupled using HCTU/6-Cl-HOBt/DIEA, while Fmoc-Lys(N_3_) was manually coupled using DIC/HOAt.

**Synthesis of tCAP(N3)**

Ac-K(N_3_)GG-PMD(NO_2_)-Gaba-NMQCQRRFYEALHDPNLNEEQRNARIRSIRDDC-NH_2_ (disulfide form)

The peptide was assembled on Rink Amide Resin (0.25 mmol) using the automated Fmoc SPPS procedure described above. The obtained resin was treated with 4-nitrophenyl chloroformate (10 eq.) in CH_2_Cl_2_ for 1 h to construct the *p*NO_2_-Phoc-MeDbz moiety [PMD(NO_2_)]. The resin was then deprotected by treatment with TFA for 1.5 h, affording the crude product, which was purified by preparative HPLC to yield the 2SH peptide (147 mg). The 2SH peptide was dissolved in AcOH/H_2_O (v/v, 1:1) and treated with 0.1 M I_2_ in MeOH at 0 °C. After stirring for 30 s, the reaction mixture was quenched with aqueous ascorbic acid and purified by preparative HPLC to yield the title peptide (87 mg, 7.3%). Analytical HPLC: *t*_R_ = 12.4 min (10–80% CH_3_CN/0.1% TFA over 25 min); purity: 98.8%.

MS (ESI, deconvoluted): calcd for C_198_H_304_N_71_O_64_S_3_ = 4798.2, found 4797.8.

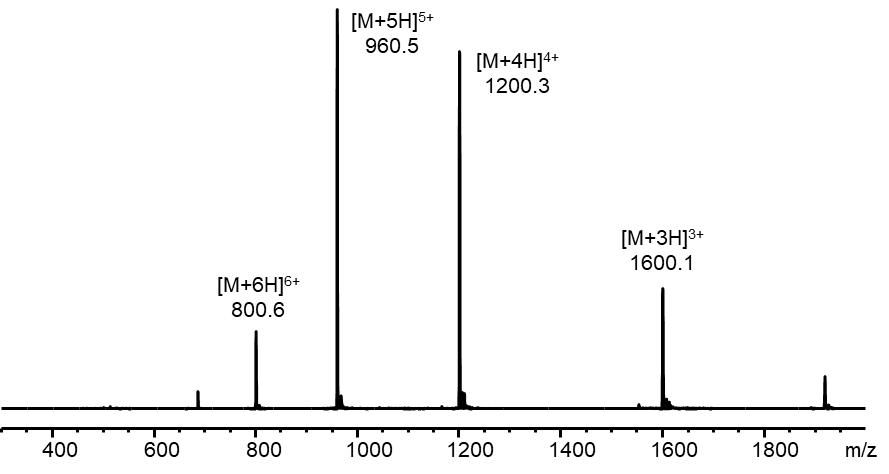


Analytical HPLC chromatogram and ESI-MS spectrum of the title peptide.

**DBCO-GGFG-Gaba-exatecan**

DBCO-Gly-Gly-Phe-Gly-Gaba-OH was assembled on Cl-Trt(2-Cl) resin (0.75 mmol) using a standard Fmoc SPPS procedure and cleaved from the resin with HFIP/CHCl_3_ (v/v, 25/75) for 2 h. The solution was concentrated *in vacuo* to give a residue (305 mg), which was used without further purification. To a solution of the residue (100 mg, 0.14 mmol, 1.2eq.) in DMF/CHCl_3_ (10 mL/5 mL), exatecan (51 mg, 0.12 mmol, 1.0 eq.), EDC (1.2 eq.), and HOBt (1.2 eq.) were added. The reaction mixture was stirred at room temperature for 17 h and then diluted with water. The crude product was extracted with EtOAc three times, and the combined organic layers were dried over MgSO_4_ and filtered. The filtrate was concentrated and purified by preparative HPLC to yield the title compound (46 mg, 34%). Analytical HPLC: *t*_R_ = 20.3/20.5 min^[40]^ (10–90% CH_3_CN/0.1% TFA for 25 min); purity: 96.0%.

MS (ESI): calcd for C_62_H_60_FN_9_O_11_ [M+H] = 1126.45, found [M+H] 1126.4.

Analytical HPLC chromatogram of the title peptide. The compound exists as a diastereomeric mixture at the DBCO moiety,^[40]^ resulting in two distinct peaks.

Reference [40]:

H. Tabata, J. Nakagomi, D. Morizono, T. Oshitari, H. Takahashi, H. Natsugari, “Atropisomerism in the Vaptan Class of Vasopressin Receptor Ligands: The Active Conformation Recognized by the Receptor” *Angew. Chem. Int. Ed.* **2011**, *50*, 3075.

**pH-Dependent Reactivity of tCAP(N3) with Tmab**

To investigate the effect of pH on the reactivity of tCAP(N3) reagent, 1.7 µL of 10 mM tCAP(N3) was mixed with 0.1 mL of 34 µM Tmab dissolved in buffers of varying pH. The following buffer systems were used: 0.1 M sodium acetate (pH 5.5), 0.1 M phosphate (pH 7.0 and 8.0), and 0.1 M sodium bicarbonate (pH 8.9). The antibody and reagent were combined at a 1:5 molar ratio and incubated for 2 h at room temperature. The reaction mixtures were then analyzed by LC–MS to evaluate the extent of conjugation.

The ratios of the different modified antibodies shown in Figure 2a were determined by simulating the peak areas of each molecular species presented in Figure 2b. First, the relative abundances (%) of the three major unmodified antibody species - *a*: 148,055; *b*: 148,218; and *c*: 148,379 - were set to a : b : c = 33.4 : 40.2 : 26.4, based on their respective peak areas. The modification efficiencies for monovalent and divalent modifications were defined as x and y, respectively. The corresponding monovalent products were denoted as *a′*, *b′*, and *c′*, while the divalent products were denoted as *a″*, *b″*, and *c″*. By varying the modification efficiencies **x** and **y** and calculating the peak ratios of the resulting species, the optimal modification efficiencies (**x** and **y**) were obtained by fitting the simulated values to the observed mass spectral area ratios using the least-squares method.

| Species | a | b | c + a’ | b’ | c’+ a’’ | b’’ | c’' |
| --- | --- | --- | --- | --- | --- | --- | --- |
| Mass | 148055-  148056 | 148218-  148220 | 148368-  148379 | 148528 | 148684-148689 | 148841-148842 | 148999 |
| Ratio  (%) | 33.4*(1-x-y) | 40.2*(1- x - y) | 26.4*(1-x-y)+ 33.4* x | 40.2* x | 26.4* x +33.4* y | 40.2* y | 26.4*y |

**Preparation of Azide-KGG–Modified Antibodies through tCAP Reaction**

For site-specific conjugation, 17 µL of 10 mM tCAP(N3) reagent was mixed with 1 mL of 34 µM Tmab in PBS at a 1:5 molar ratio of antibody to reagent. The mixture was incubated for 10 min, after which 1/10 volume of 1 M NaHCO_3_ solution (pH 8.9) was added. The reaction was then continued for 2 h at room temperature. To terminate the reaction, 1/10 volume of 10% acetic acid was added. The product was purified by dialysis overnight against a solution containing 0.1% acetic acid and 150 mM NaCl, followed by a second dialysis against PBS for 2 h to neutralize the pH. The final antibody conjugate was stored at -20 °C until further use.

**Affinity Analysis of Azide-KGG–Modified Antibodies for Fc Receptors by SPR**

The binding affinity of azide-KGG–modified antibodies to Fc receptors was evaluated at 25 °C using a Biacore 8K system (Cytiva). An anti-His tag antibody (Cytiva) was immobilized on a CM5 sensor chip by standard amine coupling, achieving a surface density of approximately 8000 RU. Fc receptors (FcγRI-His, FcγRIIa-His, FcγRIIb-His, FcγRIIIa-His, FcγRIIIb-His; Sino Biological) were subsequently captured by injection at 0.4 μg/mL, yielding ~100 RU of immobilization. Eight concentrations of azide-KGG–modified antibodies (12.5–1600 nM) were prepared in PBST buffer (PBS containing 0.005% Tween-20). For FcγRI analysis, a broader range (0.05–1600 nM; 16 concentrations) was applied. Samples were injected at a flow rate of 30 μL/min. Sensorgrams were recorded with an association phase of 150 s and a dissociation phase of 250 s (for FcγRI: 120 s association and 480 s dissociation). Binding kinetics were analyzed using the Biacore Evaluation Software with a 1:1 Langmuir binding model to determine the dissociation constant (*K*_D_).

The binding affinity of azide-KGG–modified antibodies to FcRn was evaluated similarly. FcRn (Sino Biological) was immobilized on a CM5 sensor chip by amine coupling in sodium acetate buffer (pH 5.5, Cytiva) as the immobilization solution. The receptor was injected at a concentration of 1.0 μg/mL, yielding an immobilization level of approximately 300 RU. Antibodies were injected at eight concentrations (12.5–1600 nM) in phosphate buffer (50 mM sodium phosphate, 150 mM NaCl, pH 6.0) at 30 μL/min. Sensorgrams were recorded with an association phase of 120 s and a dissociation phase of 150 s. Regeneration was performed with 100 mM Tris-HCl, 0.2 M NaCl, pH 8.0 for 30 s.

**Preparation and Validation of Tmab–Payload Conjugates via Click Chemistry**

ADCs were prepared using strain-promoted azide–alkyne cycloaddition. Azide-modified native Tmab (350 µL, 19 µM) was reacted with a threefold molar excess of each DBCO-modified payload. Specifically, 2 µL of 10 mM DBCO–Val-Cit–PAB–MMAE (BROADPHARM, San Diego, CA), 61.2 µL of 326 µM DBCO-modified hEx51_Ac0-28SN nucleic acid (custom-designed), or 2 µL of 10 mM DBCO–IRDye 800CW (LI-COR, NE, USA) was added to separate tubes containing the azide-modified Tmab. The mixtures were vortexed and incubated at room temperature for 3 h, except for the nucleic acid conjugation, which was extended to 20 h.

Similarly, 192.75 µL of 172.5 µM DBCO-modified VHH antibody specific to the IgA receptor (lab-supplied) was mixed with 350 µL of 19 µM azide-modified Tmab at a fivefold molar excess. The mixture was vortexed and incubated at room temperature for 24 h.

Following conjugation, all reaction mixtures were analyzed by liquid chromatography–mass spectrometry (LC–MS; Waters Corporation, USA) and hydrophobic interaction chromatography (HIC) to confirm successful conjugation and evaluate modification status.

**Evaluation of ADC Binding to Target Antigens by Flow Cytometry**

The experiments used SK-BR-3 cells (ATCC, HTB-30; RRID: CVCL_0033), which are HER2-positive, and C6 cells (ATCC, CCL-107), which are HER2-negative. The antigen-binding activity of four Tmab-based conjugates—Tmab–MMAE, Tmab–hEx51_Ac0-28SN nucleic acid, fluorescently labeled Tmab–IRDye 800CW, and Tmab–IgARc-L24 VHH antibody—was assessed by flow cytometry. For the binding assay, SKBR-3 cells (2 × 10⁵ cells in 100 µL of 2% FBS in PBS) were incubated with 50 ng/100 µL of each ADC. The mixtures were rotated at 4 °C for 1 h, followed by centrifugation at 200 × g for 3 min at 4 °C. After discarding the supernatant, the cell pellets were washed twice with 500 µL of 2% BSA in PBS. Detection was performed by incubating the cells with 25 ng/100 µL of pre-complexed Goat Anti-Human IgG Fc-Biotin (Abcam) and Streptavidin–Phycoerythrin (Miltenyi Biotec, Germany). The mixtures were rotated at 4 °C for 1 h, centrifuged, and washed twice with 500 µL of PBS. Finally, the cells were resuspended in 500 µL of PBS and analyzed using a flow cytometer (S3e Cell Sorter, Bio-Rad).

**Competitive Enzyme-Linked Immunosorbent Assay (ELISA) for Antigen-Binding Activity of ADCs**

Recombinant HER2-His-tag protein (25 ng/50 µL, 18.72 nM; Sino Biological Inc.) was immobilized on 96-well ELISA plates (NUNC-IMMUNO PLATE, Thermo Fisher Scientific, Denmark) and incubated at room temperature for 2 h. Wells were blocked with 0.5% BSA in PBS and stored overnight at 4 °C. After washing with PBST (PBS containing 0.1% Tween-20), 50 µL of a premixed solution containing serially diluted ADCs (ranging from 135 nM to 0.345 pM) and a fixed concentration of biotinylated Tmab (5 ng, 672 pM) in 0.01% BSA/PBS was added to each well. The plates were incubated for 1 h at room temperature with shaking. The wells were then washed three times with PBST before the addition of 50 µL of streptavidin–horseradish peroxidase (HRP) conjugate (Vector Laboratories, CA, USA). After a further 1 h incubation with shaking, wells were washed five times with PBST. Colorimetric detection was performed by adding 40 µL of TMB substrate solution, and the enzymatic reaction was allowed to proceed for 10 min. The reaction was stopped with 40 µL of 1 N HCl, and absorbance was measured at 450 nm using a microplate reader (iMark, Bio-Rad).

**Analytical FcRn Affinity Chromatography of ADCs**

Each ADC sample (10 µg in 10 µL) was analyzed by high-performance liquid chromatography (HPLC) using a Shimadzu LC2040 system (Shimadzu Corporation, Japan) equipped with a prototype analytical rFcRn column (TOSOH, Japan). This pre-packed column was designed for small-scale affinity chromatography using recombinant FcRn. Samples—including native Tmab, Tmab–tCAP, various ADCs, and monovalent/divalent Tmab–CCAP—were loaded onto the column and eluted with a gradient from 50 mM MES buffer containing 150 mM NaCl (pH 6.5) to 50 mM Tris buffer containing 150 mM NaCl (pH 8.5) at a flow rate of 0.4 mL/min.

**Analytical FcγRIIIa Affinity Chromatography of ADCs**

Each ADC sample (20 µg in 10 µL) was analyzed by HPLC using a Shimadzu LC2040 system (Shimadzu Corporation, Japan) equipped with a TSKgel rsFcR-IIIA-NPR column (TOSOH, Japan). Native Tmab, Tmab–tCAP, and various ADC samples were injected and eluted from the column with a gradient from 50 mM citric acid buffer (pH 6.5) to 50 mM citric acid buffer (pH 4.5) at a flow rate of 0.4 mL/min.

**Statistical Analysis**

Data pre-processing and basic statistical calculations (mean and standard deviation) were performed using Microsoft Excel for Microsoft 365 (version 2511). No outliers were excluded. Data are presented as mean ± SD, and the sample size (n) for each experiment is indicated in the corresponding figure legends.
